# Azoospermia and trisomy 18p syndrome: a fortuitous association? A patient report and a review of the literature

**DOI:** 10.1186/s13039-015-0141-8

**Published:** 2015-06-04

**Authors:** Guillaume Jedraszak, Henri Copin, Manuel Demailly, Catherine Quibel, Thierry Leclerc, Marlène Gallet, Moncef Benkhalifa, Aline Receveur

**Affiliations:** Médecine et Biologie de la Reproduction, Cytogénétique et CECOS de Picardie, Centre de Biologie Humaine, Centre Hospitalier Universitaire d’Amiens, Rue René Laennec, 80054 Amiens, Cedex 1 France; UFR de Médecine, Université de Picardie Jules Verne, Amiens, France; Service d’Urologie et Transplantation, Centre Hospitalier Universitaire d’Amiens, Amiens, France; Eylau/Unilabs, Paris, France

**Keywords:** Trisomy 18p, Azoospermia, Fertility disorder, Meiotic synapsis defect

## Abstract

Complete, isolated trisomy of the short arm of chromosome 18 is very rare. To date, only 24 cases of trisomy 18p have been reported in the literature, making it difficult to define a potentially associated phenotype. However, the available evidence suggests that few clinical features are shared by these patients: only variable intellectual disability, variable facial dysmorphism and epilepsy are reported in a few patients. Although three inherited cases of trisomy 18p have already been reported, all were of maternal origin.

We report on a patient carrying an isolated complete trisomy 18p translocated to the short arm of chromosome 14 and presenting with facial dysmorphism, mild intellectual disability and non-obstructive azoospermia.

Chromosomal abnormalities are more frequent in infertile men with poor sperm quality than the general population. Both numerical and structural chromosomal aberrations have been already reported within the context of azoospermia. To our knowledge, this is the first patient with trisomy 18p to present a fertility impairment due to totally altered spermatogenesis and azoospermia. Although fertility disorders were not mentioned in the four previous reports of men with trisomy 18p, none of the latter had children. We suggest that azoospermia is a previously uncharacterized feature of trisomy 18p syndrome. We further hypothesize that two mechanisms could be responsible of the fertility impairment: a meiotic synapsis defect due to the additional 18p arm that blocks meiosis, and/or overexpression of a gene located on the 18p chromosome involved in the normal testicular development.

## Background

Complete, isolated trisomy of the short arm of chromosome 18 is very rare. To date, only 24 such patients have been described in the literature (for a review, see [1]). Although these individuals presented with variable facial dysmorphism and possible mild to moderate cognitive development delay, there are no specific shared clinical features. Four of the 24 patients were adult males. Although fertility disorders were not mentioned in these four reports, none of the latter declared pregnancies and had children, suggesting that a spermatogenetic defect can be associated to trisomy 18p syndrome.

Infertility is a common impairment: approximately 15 % of couples are unable to conceive after one year of unprotected intercourse. A male factor (mostly abnormal semen parameters) is solely responsible in 20 % to 30 % of these cases [2]. Chromosomal abnormalities are more frequent in infertile men with poor sperm quality than the general population [3]. Some of these abnormalities can affect spermatogenesis efficiency and disturb semen parameters, and increase the risk of recurrent miscarriage or congenital anomalies. As a consequence, international guidelines state that infertile men must be screened for chromosomal abnormalities before starting on an assisted procreation program [3].

We report on a patient presenting with facial dysmorphism, mild intellectual disability and non-obstructive azoospermia. The results of cytogenetics analyses (karyotyping, AZF microdeletion research and array comparative genomic hybridization (CGH)) allowed us to diagnose isolated, complete trisomy 18p translocated to the chromosome 14p region. To our knowledge, this is the first report of a patient with a nonobstructive azoospermia possibly caused by an alteration of the spermatogenesis, associated with trisomy 18p.

## Case Presentation

### Patient report

**Fig. 1:**
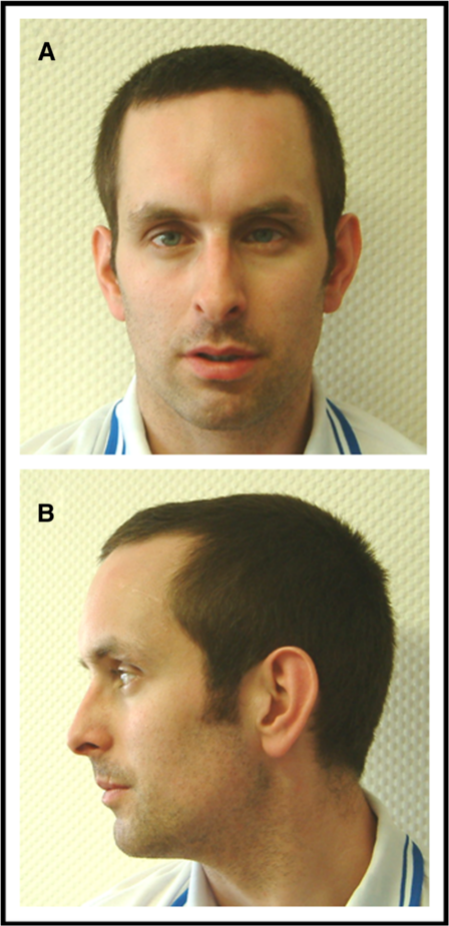
Facial dysmorphism. Facial dysmorphism of our patient in a frontal (**a**) and lateral (**b**) view. Facial dysmorphism consisted of high and narrow forehead, hypotelorism, deep-set eyes and abnormal ears

A 28 year-old male was referred to our Reproductive Biology Centre for investigation of primary infertility. He was the first child of unrelated, healthy parents. There was no familial history of malformation, intellectual impairment or fertility disorders. The patient had one sister and one brother who had both healthy children. He had been born after an uneventful pregnancy. At birth, weight was 3500 g, height was 49 cm and head circumference was 37 cm. The patient’s personal medical history was marked by a hearing impairment (due to recurrent otitis and to a cholesteatoma requiring a surgical treatment) and strabismus (also requiring a surgical treatment). He had no medical history of a urogenital pathology (congenital malformation, cryptorchidism, varicocele, testicular torsion, tumor, *Myxovirus parotidis* infection, inguinal hernia), or exposure to factors that may harm spermatogenesis. There had not been any delay in psychomotor development: the patient had been able to sit from the age of six months and first walked at the age of fourteen months. We noted a mild intellectual disability: the patient lives independently, has a job, but presents difficulties in reading and writing; he had attended a specialized school and is not able to be graduated. Upon clinical examination, we noted discrete facial dysmorphism (Fig. 1) and a slight bilateral hypotrophy of testicles, and secondary sex characteristics had developed normally. The patient was 180 cm tall (50^th^-75^th^ percentile) and weighed 72 kg (50^th^ percentile). The facial dysmorphism consisted of a high, narrow forehead, hypotelorism, deep-set eyes, and abnormally shaped ears (Fig. 1). An examination of the hands revealed long fingers and fifth finger clinodactyly. Male infertility was investigated in accordance with the international guidelines [3] and semen analysis were performed in accordance with the WHO guidelines (2010). Semen analyses revealed normal physical parameters (ejaculate volume 2 ml, pH 8.1, normal viscosity) and azoospermia (confirmed after the centrifugation of two different samples). Levels of testosterone were normal (3.74 ng/ml – normative values 2,8 to 8 ng/ml) and follicle-stimulating hormone (24.2 IU/L – normative values 2–15 UI/L) and luteinizing hormone (13.1 UI/L – normative values in our laboratory 1,7 to 8,6 UI/L) levels were elevated, suggesting nonobstructive azoospermia. Testicular ultrasonography was normal and testicular biopsy showed severe altered spermatogenesis (only few spermatozoa in less than 20 % of seminiferous tubules on the right testicular biopsy, and few spermatozoa in less than 10 % of seminiferous tubules on the left testicular biopsy) which explained the azoospermia and infertility.

### Cytogenetic analyses

Cultured peripheral blood lymphocytes were karyotyping using Reverse-Heat-Giemsa (RHG) and Giemsa-Trypsine-Giemsa (GTG) banding according to standard methods. Fluorescence in-situ hybridization (FISH) analyses with 18p and 18q telomere probes (Abbott Molecular Diagnosis, Abott Laboratories, Illinois, USA) were performed according to the manufacturer’s protocols. In order to characterize the chromosome anomaly, 44 K array-CGH (AgilentTM, Agilent Technologies, Santa Clara, CA, USA) was performed according to standard protocols and the manufacturer’s recommendations.

**Fig. 2:**
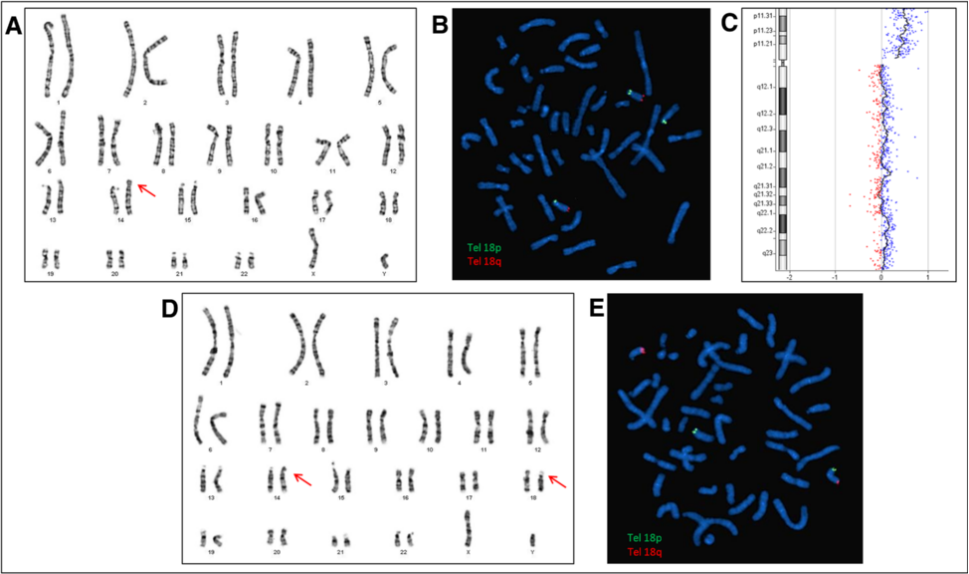
Cytogenetics analyses. Patient’s karyotyping (**a**) revealed an additional segment on the short arm of one chromosome 14 (red arrow), which is labeled by a telomere 18p arm FISH probe (**b**). Array-CGH results showing a pure and complete trisomy 18p (**c**). It is the result of the unbalanced inheritance of a paternal t(14;18)(p10;p10) (**d** and **e**)

The cytogenetics analyses revealed an additional segment on the short arm of one chromosome 14 (Fig. 2a). The array CGH results showed the presence of isolated, complete trisomy 18p (Fig. 2c). This result has been confirmed by FISH analyses (Fig. 2b). Karyotype and FISH analyses (telomere 18p and 18q probes) were normal for his mother, and showed a balanced translocation t(14;18)(p10;p10) for his father (Fig. 2d and e). Patient’s final karyotype was: 46,XY,der(14)t(14;18)(p10;p10)pat.arr 18p11.32p11.21(14,316-14,733,870)(hg19)x3

Common Y microdeletions were ruled out by studying of six markers of the AZF region (sY84 and sY86 in the AZFa region, sY127 and sY134 in the AZFb region and sY254 and sY255 in the AZFc).

## Discussion

Isolated trisomy 18p is a rare anomaly; only 24 patients with this condition have been described in the literature [1, 4–18]. Our patient is the fifth male adult reported to date. He presented with facial dysmorphism, a mild intellectual disability and a sign that has never previously been reported in this context: non-obstructive azoospermia. AZF region microdeletion was ruled out. Hence, this is the first report of a fertility disorder due to alteration of the spermatogenesis associated with isolated trisomy 18p.

Infertile men with poor sperm quality are more likely to carry chromosomal abnormalities than the general population, with an inverse correlation between chromosomal abnormalities and sperm concentration [19]. A recent study found abnormal karyotypes in 15.2 % of infertile men with azoospermia [20]. The most common genetic abnormalities found in infertile men with non-obstructive azoospermia are related to numerical and structural chromosomal aberrations. They include Klinefelter syndrome or chromosomal translocation that impair testicular function, and Y-chromosome microdeletions that are associated with isolated defects in spermatogenesis [21].

Several recurrent cytogenetic mechanisms underlying trisomy 18p syndrome have been described (for a review, see [1]). Our patient is the third case of isolated, complete trisomy 18p caused by an unbalanced translocation involving the chromosome 14p [11, 13]. Furthermore, he is the first to have been investigated with array-CGH, which (i) confirmed that the entire 18p arm was involved and (ii) ruled out any other chromosome imbalance.

The low number of reported cases of trisomy 18p syndrome makes it difficult to define a potentially associated phenotype (for a review, see [1]). The most commonly reported features are mild to moderate intellectual disability or developmental delay (15 out of 24 patients), epilepsy (4 out of 24 patients) and non-specific dysmorphic features. In contrast, azoospermia has never been reported. Four male adults, who were between 21 and 37 years, have been described to date [1, 10, 12]; none of the corresponding reports mention paternity. Furthermore, inability to conceive was the only impairment presented by one of these patients (case 5 in [10]). Although three inherited cases of trisomy 18p have been reported, all were of maternal origin (two cases of duplication of 18p arm [6, 7] and one case with transmission of a small supernumerary marker chromosome [16]). These data are consistent with possible male infertility associated with trisomy 18p syndrome.

According to the UCSC Genome Browser (http://genome.ucsc.edu/), 66 RefSeq coding genes are included in the duplicated region. Despite the fact that the addition of a complete arm on an acrocentric chromosome can directly affect spermatogenesis by inducing defects in meiotic synapsis and disturbing the meiotic behavior of chromosomes [22] , some triplicated genes could also be involved in fertility disorders. Two of them have been studied in spermatogenesis in mice: *LAMA1*, which encodes one of the laminin alpha chain, a protein involved in the membrane basement of the seminiferous epithelium and required for normal testicular function [23]; and *TXNDC2*, which encodes a sperm-specific protein that is an important regulator of critical steps in human spermatogenesis [24, 25]. However, there are no specific data on a possible pathogenic effect of the deregulation of these genes on the human spermatogenesis; these hypothesis need to be confirmed by further studies.

## Conclusion

In conclusion, we report on the first patient with complete, isolated trisomy of the short arm of chromosome 18 due to an unbalanced translocation involving chromosome 14p and confirmed by array CGH. The patient presented with a mild intellectual disability and a feature that has never previously been reported in trisomy 18p syndrome: a non-obstructive azoospermia. The additional short arm of chromosome 18 on an acrocentric chromosome could probably induce a meiotic synapsis defect, but we can’t exclude a potential additive effect of a triplicated gene, such as *LAMA1* and *TXNDC2*, in the spermatogenesis defect. We hypothesize that azoospermia is a previously unknown feature associated with trisomy 18p syndrome.

## Consent

Written informed consent was obtained from the patient for publication of this case report and any accompanying images. A copy of the written consent is available for review by the Editor-in-Chief of this journal.

## Abbreviations

CGH: Comparative genomic hybridization
FISH: Fluorescence in-situ hybridization
AZF: AZoospermia factor
